# The role of microRNAs in nicotine signaling

**DOI:** 10.17179/excli2023-6096

**Published:** 2023-05-19

**Authors:** Khalil Hajiasgharzadeh, Bahman Naghipour, Parviz Shahabi, Narges Dastmalchi, Mohammad Reza Alipour

**Affiliations:** 1 Stem Cell Research Center, Tabriz University of Medical Sciences, Tabriz, Iran; 2 Department of Anesthesiology, Faculty of Medicine, Tabriz University of Medical Sciences, Tabriz, Iran; 3 Department of Physiology, Faculty of Medicine, Tabriz University of Medical Sciences, Tabriz, Iran; 4 Department of Biology, University College of Nabi Akram, Tabriz, Iran

**Keywords:** cigarette smoking, nicotine, nicotinic acetylcholine receptors, microRNA, signaling pathway

## Abstract

Cigarette smoking is a harmful habit that is widespread around the world. It is among the well-known lifestyle-related risk factors for many diseases. Nicotine, as its principal constituent, has various detrimental, and beneficial functions. Nicotinic acetylcholine receptors (nAChRs), which are present in nearly all body cells, are how nicotine works. Numerous investigations have demonstrated that nicotine causes abnormal microRNA expression (miRNAs). These short sequences of RNAs are known to regulate gene expression post-transcriptionally. A wide range of miRNAs are modulated by nicotine, and nicotine-induced miRNA changes could subsequently mediate nicotine's effect on gene expression regulation. We will focus on the reciprocal interaction between nAChRs and miRNAs and describe the essential targets of these dysregulated miRNAs after nicotine exposure and activation of nAChRs. It appears that crucial subcellular mechanisms implicated in nicotine's effects are miRNA-related pathways. It is crucial to investigate the molecular mechanism underlying the effects of nicotine as well as the dysregulation of miRNA following nAChR activation. The finding about epigenetic mechanisms of nicotine-induced effects may shed light on the establishment of new treatment strategies to prevent the harmful effects of nicotine and perhaps may augment the beneficial effects in diverse smoking-related diseases.

## Introduction

Smoking is one of the most important risk factors for many human diseases, including cancers, chronic obstructive pulmonary disease, cardiovascular disease, and type 2 diabetes mellitus (Rigotti et al., 2022[62]). Nicotine, an essential ingredient of cigarette smoke, binds to the nicotinic acetylcholine receptors (nAChRs) and induces many downstream signaling pathways (Madsen et al., 2015[45]; Cuevas‐Olguin et al., 2020[12]; Pucci et al., 2021[56]). Recent studies have demonstrated that nicotine modifies the expression of microRNAs (miRNAs) in many smoking-related disorders and exerts its effects through miRNA-related pathways (Mullany et al., 2016[49]; Civelek, 2017[11]). miRNAs are endogenous non-coding RNAs that modulate the expression of numerous genes (Dastmalchi et al., 2022[16]). These molecules are crucial participants in many physiological and pathological conditions. Various studies showed that smoking leads to changes in the expression of miRNAs. Cigarette smoking substantially alters miRNA profiles in healthy ones (Takahashi et al., 2013[75]). Regarding the prevalence of this habit which has become a frontier topic and research hotspot, many researchers have tried to identify the underlying molecular mechanisms to combat its side effects. But the significance of these perturbations in humans remains to be understood in detail. The present review study aimed to highlight the miRNA-based molecular mechanism underlying nicotine-induced adverse effects. We have highlighted all the evidence from animal and human studies that shows the relationship between nicotine administration, and the function of nAChRs with changes in the expression of miRNAs. This article examines the role of miRNA in nicotine exposure and smoking. This review essay offers a fresh viewpoint on the literature because there hasn't been a current review of miRNAs in nicotine signaling. Further studies on this topic can lead to finding promising therapeutic targets for nicotine-induced diseases.

## Nicotine and Its Receptors

**Figure 1:**
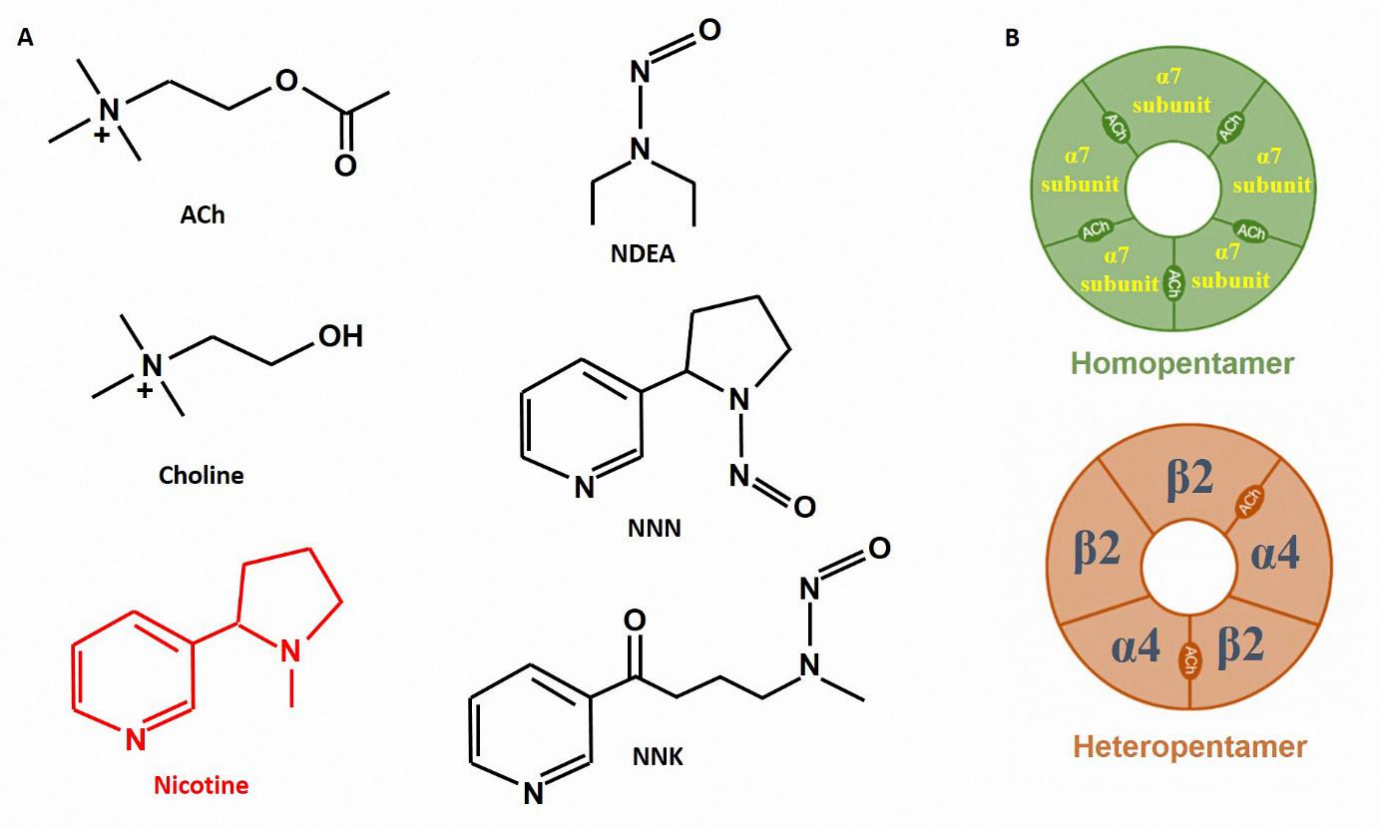
(A) Chemical structures of acetylcholine (ACh), choline, nicotine, and three tobacco smoke-related N-Nitrosamines, including N-Nitrosodiethylamine (NDEA); N‐Nitrosonornicotine (NNN); and 4‐(methylnitrosamino)‐1‐(3‐pyridyl)‐1‐butanone (NNK). All of these compounds can bind and activate nicotinic receptors. (B) These nicotinic receptors can be homopentamer or heteropentamer. They are the assembly of five subunits that are arranged around a central I, on pore.

Smoking habit is one of the chief modifiable risk factors for many smoking-induced disturbances (Phillips and Glover, 2022[52]). Smoking is highly prevalent worldwide, and about 22 % of people worldwide smoke tobacco, according to World Health Organization research (WHO, 2017[87]). Nicotine is a significant component of tobacco smoke, even though it contains over 5,000 other chemicals. Nicotine, as a main alkaloid compound present in smoking (about 95 % of the total alkaloid fraction) alone or as a component of tobacco smoke, has a fundamental role in the initiation and development of many diseases via binding to nAChRs (Rodgman and Perfetti, 2008[63]; Hajiasgharzadeh et al., 2020[30]). The list of diseases connected to nicotine is expanding. This substance is a bioactive molecule that acts upon nAChRs expressed in many different cells and tissues of the body. It was demonstrated that nAChR, which is involved in the regulation of intracellular processes including cytochrome c release, is expressed by intracellular organelles such as mitochondria in addition to the plasma membrane (Skok, 2022[70]). On the other hand, in addition to nicotine, many other endogenous nAChRs agonists, such as acetylcholine and choline as well as other exogenous compounds such as tobacco smoke N-Nitrosamines can bind to nAChRs and activate them (Afrashteh Nour et al., 2021[1]) (Figure 1A(Fig. 1)). In vertebrates, 17 different subunits, including (α1-10, β1-4, δ, ε, γ) have been identified for nAChRs, which are placed together as homopentamer or heteropentamer and form a large family of different types of receptors (Dani and Bertrand, 2007[13]) (Figure 1B(Fig. 1)). Several studies confirmed that first-hand or second-hand exposure to nicotine upregulates nAChRs (Govind et al., 2009[27]; Hajiasgharzadeh et al., 2020[29]). Moreover, there are many findings about the diverse effects of nicotine via these receptors in most tissues. Alpha-7 nicotinic acetylcholine receptors (α7nAChR), among well-known nAChRs, are a family of ligand-dependent ion channels, which are made as a homopentamer of five alpha-7 subunits (Hajiasgharzadeh et al., 2019[28]). These receptors are expressed by the CHRNA7 gene located on chromosome 15q14, and its final product is a protein with a molecular weight of 55 kilo Daltons (Tracey, 2009[78]). They are expressed in almost all cells of the body and play a variety of roles in the cells. Several studies demonstrated the interaction between α7nAChR, and miRNAs (Winek et al., 2021[85]). For instance, miRNA-98 downregulation increased α7nAChR expression (Song et al., 2021[72]). In another paper, Sun et al. indicated that nicotine could inhibit signal transducer and activator of transcription 3 (STAT3) activity by inducing miRNA-124 via α7nAChR (Sun et al., 2013[74]). One of the main ingredients in cigarettes, nicotine serves both harmful and advantageous purposes. These contentious results might be attributed to the various *in vivo *and* in vitro* models. Furthermore, a consistent association between nicotine and its receptors and miRNA alteration was confirmed. However, the exact details about the molecular mechanism of the various effects of nicotine are unclear. Taken together, the physiological effects of nicotine are mediated through binding to and activation of nAChRs (Egleton et al., 2008[22]; Dasgupta et al., 2009[14]; Schuller, 2009[65]). But the exact intracellular signaling pathways of these receptors and the participation of miRNAs in them remain to be identified.

## miRNA

**Figure 2:**
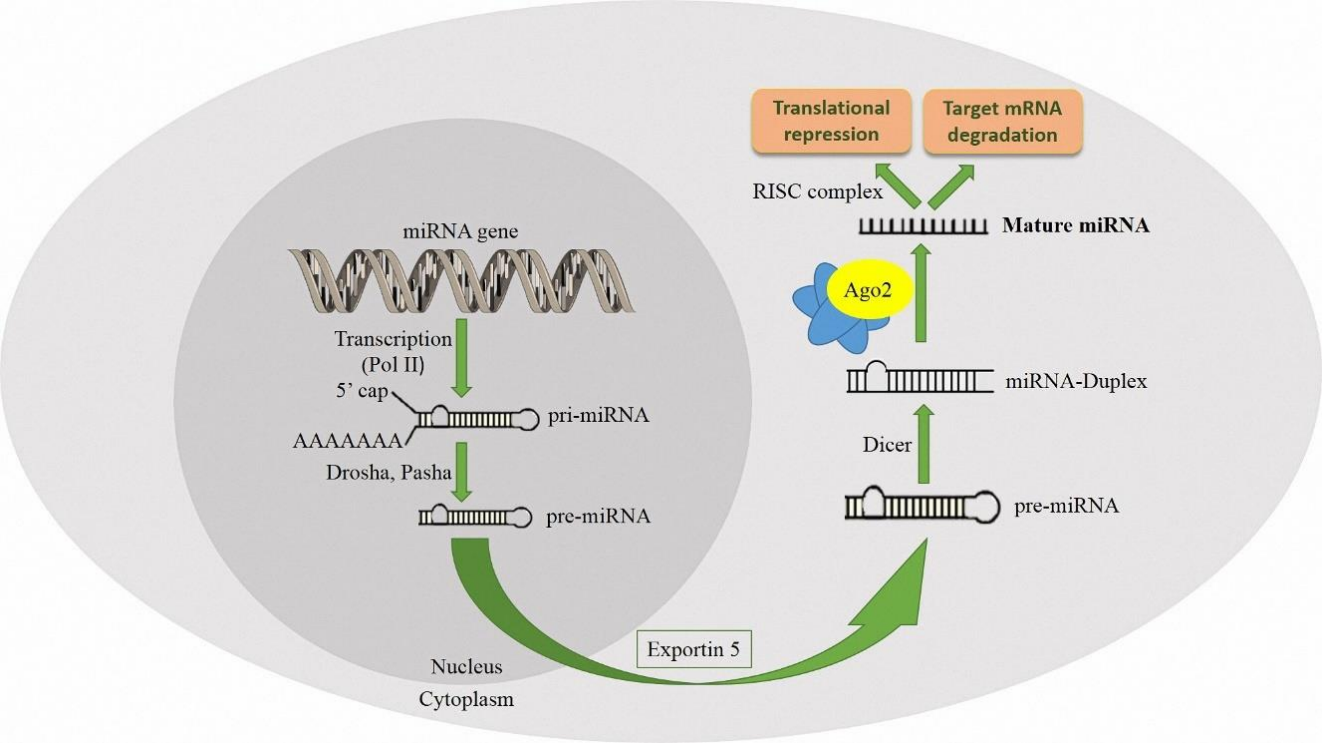
miRNAs are transcribed by RNA polymerase II to primary-miRNA (pri-miRNA) that can be cleaved by Drosha and Pasha in the nucleus. This miRNA is then processed into precursor RNA (pre-miRNA). Then, by Exportin 5, the pre-miRNA is exported to the cytoplasm and further processed by Dicer, to produce a duplex miRNA. Finally, the mature miRNA is formed and binds to an Ago protein and incorporates into the RNA-induced silencing complex (RISC). This mature miRNA causes translational repression and target mRNA degradation.

miRNAs are evolutionarily conserved non-coding RNA sequences with about 22 nucleotides length, expressed in plants, animals, and some viruses (Zeinali et al., 2020[91]). These molecules, either by translational repression or by mRNA degradation, play an essential role in RNA control, post-transcriptional regulation, and gene expression (Fabian and Sonenberg, 2012[24]). The human genome encodes around 2,000 microRNAs, the majority of which have been discovered so far (Alles et al., 2019[2]). Consequently, they regulate numerous genes involved in diverse biological processes, such as cell development, cell death, metabolism, immune responses, and several other cellular functions and signaling pathways (Xiao et al., 2016[89]). Many studies emphasize how the miRNA-mediated silencing complex controls gene expression (Cai et al., 2009[7]; Vishnoi and Rani, 2023[82]). In brief, miRNAs are transcribed by RNA polymerase II (Pol II) to primary-miRNA (pri-miRNA) that can be recognized and cleaved by ribonucleases Drosha and Pasha in the nucleus (Tomankova et al., 2010[77]). These miRNAs are then processed into precursor miRNA (pre-miRNA). Then, by Exportin 5, the pre-miRNA is exported to the cytoplasm and processed by the Dicer enzyme, to produce a duplex miRNA (Komatsu et al., 2023[35]). The mature miRNA is formed and binds to an Ago protein and incorporates it into the RNA-induced silencing complex (RISC). miRNAs act based on the base pair relationship with their complementary sequences within the mRNA molecules. The pre-miRNA has two components: miRNA-5p and miRNA-3p, and depending on the organ, both components can become functional (Mitra et al., 2015[48]). It regulates downstream target genes' expression by binding with the 3' untranslated region (3'UTR) of the target mRNA. Finally, mature miRNA causes translational repression and targets mRNA degradation (Dastmalchi et al., 2020[15]) (Figure 2(Fig. 2)). 

## Nicotine Exerts Its Effects through the miRNA Pathway

Dysregulation of miRNAs in terms of the activation of nAChRs has been associated with the pathogenesis of several diseases. Increasing evidence showed the changes in the expression of miRNAs following nicotine supplementation and its correlation with the expression level of nAChRs. In a recent study, Lallai et al. showed that nicotine modulates miRNA-204 expression profiles to influence cholinergic signaling and numerous nAChR subtypes (Lallai et al., 2019[37]). They showed that nicotine administration in rats increased expression of miRNA-204 and nAChRs in the choroid plexus of the brain in similar expression profiles and revealed the direct mechanism by which nicotine modulates the function of this tissue via miRNA-204/nAChRs axis (Lallai et al., 2019[37]). Takahashi et al. studied whether the expression of miRNAs in healthy people changes with smoking cigarettes. They found that 44 detected miRNAs were significantly higher in repeated cigarette smoking subjects in comparison to non-smoker ones. Among these miRNAs, 24 of them were previously reported to be potential biomarkers of disease, suggesting the possibility of the application of these miRNAs as candidate biomarkers and highlighting that the smoking habit might interfere with the diagnosis of disease (Takahashi et al., 2013[75]). The specific expression profiles of miRNAs may be altered in terms of external factors such as exposure to nicotine and subsequently, may cause alteration in tissue functions.

Balaraman et al. designated that the observed effects of nicotine on miRNA expression are due to the function of nAChRs in neural stem cells (Balaraman et al., 2012[4]). They showed that nicotine increased the expression of α4 and β2 nAChR transcripts. Moreover, at concentrations observed in cigarette smokers, nicotine disrupts several miRNA regulatory networks, including miRNA-9, miRNA-21, miRNA-153, miRNA-335, and miRNA-140-3p expression. These effects were blocked by an nAChRs antagonist, which showed that the effects of nicotine on miRNAs' expression were nAChR dependent (Balaraman et al., 2012[4]). These data indicated that nicotine-altered miRNA expression is essential for neural stem cell maturation, neural differentiation, synaptogenesis, inflammation, memory, and cognition (Balaraman et al., 2012[4]). In addition, Taki and colleagues demonstrated that chronic nicotine intake drastically altered the miRNA expression profiles and their associated signaling pathways in a model organism (Taki et al., 2014[76]). According to their findings, nicotine exposure affected the expression of 40 microRNAs in a dose-dependent manner. This study provided new insight for a better understanding of the effects of nicotine in early developmental stages. In another study, Banerjee et al. tried to quantify the plasma miRNAs in healthy smokers, ex-smokers, and non-smokers (Banerjee et al., 2015[5]). The results indicated the differential expression of miRNA-124 and let-7a between the smoking, and non-smoker groups. Thus miRNA-124 and let-7a could be promising biomarkers of biological effects after cigarette smoke exposure (Banerjee et al., 2015[5]). Nicotine might be correlated with the expression changes of different miRNAs. Thus, in the canines model of atrial fibrillation, nicotine produced a significant decrease in the levels of miRNA-133 and miRNA-590 which indicates that the effects of nicotine are dependent on these two miRNAs (Shan et al., 2009[67]). 

Recently, it was discovered that cigarette smoke significantly downregulates 24 miRNAs in the lungs of rats (Du et al., 2018[20]). Du et al. studied the processes underlying nicotine-induced periodontitis and the miRNA expression profile of human periodontal ligament (PDL) cells exposed to nicotine (Du et al., 2019[19]). 

**Figure 3:**
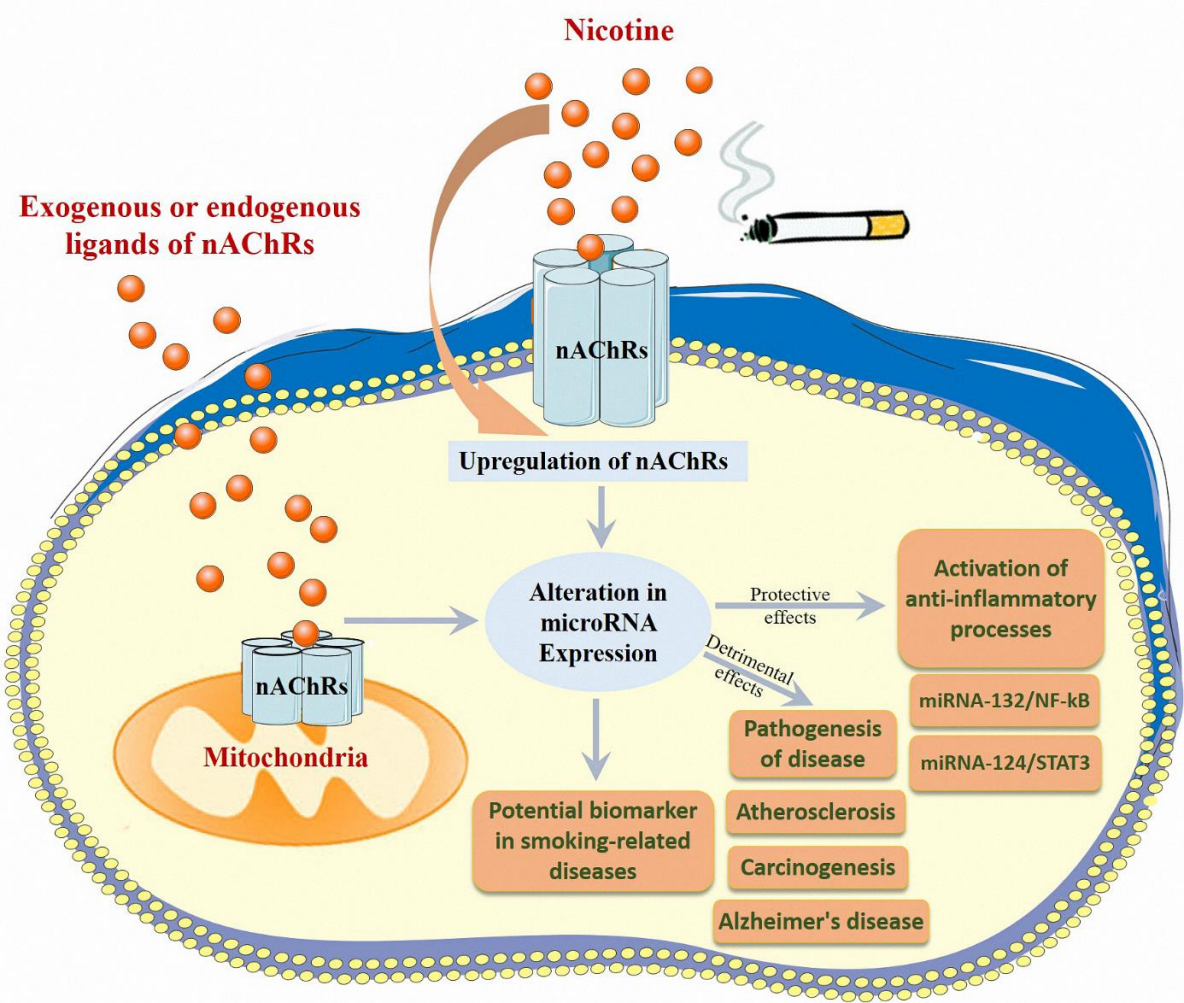
This review covers many different miRNAs that their expression changes due to nicotine exposure. We highlighted the crosstalk between deregulated miRNAs caused by exposure to nicotine or other exogenous or endogenous ligands of nAChRs and their subsequent outcomes.

Further analysis of target genes of these dysregulated miRNAs indicated that several critical signaling pathways, such as the dynamin 1 signaling pathway, transforming growth factor beta (TGF-β) signaling pathway, the fos-1 signaling pathway, and the phosphatase and tensin homolog (PTEN) signaling pathway, are potentially responsible for nicotine-induced diseases (Table 1(Tab. 1); References in Table 1: Balaraman et al., 2012[4]; Du et al., 2018[20]; Gomez et al., 2016[26]; Huang and Li, 2009[33]; Liu et al., 2015[41], 2019[43], 2022[42]; Maegdefessel et al., 2012[46]; Pittenger et al., 2018[54]; Qin et al., 2020[59]; Rauthan et al., 2017[61]; Shin et al., 2011[68]; Shrestha et al., 2020[69]; Solleti et al., 2017[71]; Song et al., 2021[72]; Taki et al., 2014[76]; Wongtrakool et al., 2020[86]; Wu et al., 2020[88]; Zhang et al., 2014[92], 2022[93]; Zhu et al., 2019[94]). Another study by Wasén and colleagues indicated that smoking changed the miRNA profile, and smokers were recognized by differential expression of 8 miRNAs (Wasén et al., 2020[84]). In this study on CD8+ cells, the results showed that miRNAs involved in the FOXO-signaling pathway, including let-7c-5p, let-7d-5p and let-7e-5p, miRNA-92a-3p, miRNA-150-5p, and miRNA-181-5p were upregulated, while miRNA-3196 and miRNA-4723-5p were downregulated (Wasén et al., 2020[84]). Recently Ahmad Khan et al. studied the miRNA expression patterns in response to cigarette smoke and chewing tobacco and identified several miRNAs that showed significantly altered expression in cigarette smoke-exposed cells (Khan et al., 2018[34]). In the Liu et al. experiment, mice were subjected to nicotine treatment and the results showed that nicotine suppressed let-7c-5p (Liu et al., 2022[42]). According to the findings of earlier studies, miRNA-4466 may serve as a possible biomarker for predicting an increased risk of metastatic illness among smokers (Tyagi et al., 2022[80]). Despite all of this research, further attention must be paid to the specific effects of nicotine and its receptors on microRNA pathways. Studies on cell- and tissue-specific miRNA changes will allow us to better understand the role of epigenetic changes in the development of diseases (Figure 3(Fig. 3)). In the following subsections, we will discuss these studies that highlighted the modulatory roles of miRNAs in the observed effects of nicotine and nAChRs.

### miRNA-16

Numerous findings indicated that smoking changed the function of the cells via a miRNA-dependent mechanism. Shin et al., using a miRNA array platform, investigated 95 human miRNAs to explore the expression profile in nicotine-treated cultured gastric cancer cells (Shin et al., 2011[68]). They found that miRNA-16 was upregulated upon nicotine stimulation (Shin et al., 2011[68]). Nicotine, in a dose-dependent manner, leads to the degradation of the inhibitor of kappa B and induced the nuclear factor-kappa B (NF-kB) translocation. Further studies indicated that nicotine induced the binding of NF-kB to the promoters of miRNA-16. These results suggest that nicotine-promoted miRNA-16 and some other miRNAs expression are modulated by the transcription factor NF-kB (Shin et al., 2011[68]).

### miRNA-21

Since miRNAs are essential regulators of cardiovascular disease, in murine models of vascular abnormality, Maegdefessel et al. indicated that nicotine supplementation in concentrations similar to those present in the vascular tissue of medium to heavy smokers upregulates miRNA-21 expression and induced pro-proliferative and antiapoptotic activities. In this work, miRNA-21 increased cell proliferation and decreased apoptosis in the aortic wall, hence inhibiting aneurysm growth. Increased expression of miRNA-21 dramatically decreased expression of the PTEN protein, resulting in increased AKT protein activation. They conclude that nicotine-induced expression of miRNA-21 and inhibition of PTEN activity via miRNA-21 may be novel therapeutic methods to treat vascular abnormalities (Maegdefessel et al., 2012[46]). 

Zhang et al. showed that upregulation of miRNA-21 is associated with cigarette smoking as well as nicotine treatment. They studied the role of nicotine-induced miRNA-21 expression in the epithelial-to-mesenchymal transition of esophageal cancer cells. They found that nicotine-induced miRNA-21 promotes TGF-β activity (Zhang et al., 2014[92]). Their study reveals that nicotine functions in cancer cells via the miRNA-21/TGF-β pathway. Similarly, Zhu et al. showed that exosomal miRNA-21 from nicotine-treated macrophages might accelerate the development of atherosclerosis by increasing vascular smooth muscle cell migration and proliferation through its target PTEN (Zhu et al., 2019[94]). Zhang et al. studied the molecular mechanism underlying nicotine-induced chemoresistance in lung cancer (Zhang et al., 2022[93]). qRT-PCR techniques were used to evaluate the expression of miRNA-21 and its target gene in the presence or absence of nicotine (Zhang et al., 2022[93]). The luciferase reporter tests demonstrated that miRNA-21 and FOXO3 interact. Nicotine induced miRNA-21 expression in lung cancer cells in a dose-dependent manner. Subsequently, miRNA-21 downregulated FOXO3a expression by directly binding to the 3'‑untranslated region of FOXO3a and promoted chemoresistance to standard chemotherapy drugs (Zhang et al., 2022[93]).

### miRNA-24

Ebrahimpour et al. showed that nicotine modulates miRNA-24 as an anti-inflammatory miRNA, and its downstream targets promote inflammatory and fibrotic functions in lung tissue (Ebrahimpour et al., 2019[21]). This miRNA was suppressed by nicotine during lung injury. Moreover, they found that nicotine upregulates the expression of inflammatory cytokines targeted by miRNA-24 (Ebrahimpour et al., 2019[21]).

### miRNA-30

Contrary to research showing that nicotine increases cell proliferation, several other investigations have found that nicotine also inhibits the proliferation of some types of cells (Du et al., 2019[19]). Many studies showed that nicotine inhibits the regeneration of periodontal tissues primarily by blocking the proliferation of human PDL cells (Chang et al., 2001[9], 2002[8]). Wu et al. showed that nicotine-upregulated miRNA-30a expression (Wu et al., 2020[88]). This upregulated miRNA-30a expression subsequently blocks the proliferation of human PDL cells by downregulating the expression of cyclin E2. The inhibition of miRNA-30a restored cyclin E2 expression that had been downregulated by nicotine (Wu et al., 2020[88]).

### miRNA-98

Post-transcriptional regulation of α7nAChR expression by miRNA-98-5p was studied in a recent innovative study (Song et al., 2021[72]). Song et al. showed a key regulatory mechanism of α7nAChR expression in Alzheimer's disease and further showed that miRNA-98-5p inhibited α7nAChR expression through directly binding to 3′UTR of mRNA (Song et al., 2021[72]). Additional *in vitro *and* in vivo* experiments demonstrated that suppression of miRNA-98-5p causes enhanced α7nAChR expression and ameliorated neuroinflammation via suppressing the NF-kB pathway and upregulating Nrf2 target genes. *In vitro* lung fibroblasts and *in vivo* lung homogenates, nicotine decreased the levels of miRNA-98, according to research by Wongtrakool and colleagues (2020[86]). This event subsequently increased several target genes involved in asthma development. Their findings showed that nicotine-induced increases in nerve growth factor and other markers of airway remodeling are negatively regulated by miRNA-98 (Wongtrakool et al., 2020[86]).

### miRNA-126

The effect of nAChR stimulation with nicotine on the regulation of miRNA expression and subsequent molecular pathways was investigated in different studies. Sugiura et al. showed the effects of cigarette smoking and nicotine exposure on vascular endothelial damage and the pathogenesis of atherosclerosis in smokers (Sugiura et al., 2015[73]). They evaluated the expression of miRNA-126 and discovered that quitting smoking decreased endothelial damage and boosted plasma levels of circulating miRNA-126. They concluded that miRNA-126 might serve as a biomarker for recovery from smoking-related vascular injury (Sugiura et al., 2015[73]). In addition to conventional cigarettes, electronic cigarettes have increased usage (Kopa-Stojak and Pawliczak, 2023[36]). Recently Kopa-Stojak and Pawliczak conducted a systematic review analysis to compare the effects of tobacco cigarettes, electronic nicotine delivery systems, and tobacco heating products on miRNA-mediated gene expression. They showed that the altered expression of miRNAs was reduced in electronic cigarettes and tobacco heating product users in comparison to cigarette smokers (Kopa-Stojak and Pawliczak, 2023[36]). However, the expression of some miRNAs was significantly altered in both electronic cigarettes and tobacco heating products compared to air controls (Kopa-Stojak and Pawliczak, 2023[36]). Additionally, Solleti and associates looked into how e-cigarettes affected the expression of miRNA in human lung epithelial cells (Solleti et al., 2017[71]). They discovered that exposure to electronic cigarettes results in the expression of 578 miRNAs being dysregulated. Further studies validated the enhanced expression of multiple miRNAs, including miRNA-126, after electronic cigarette exposure. Furthermore, additional mechanistic experiments revealed that the expression of MYC and MRGPRX3a as two essential miRNA-126 target genes was significantly reduced (Solleti et al., 2017[71]).

### miRNA-132

Another study identified a new miRNA-based mechanism through which miRNA-132 modulates autoimmune encephalomyelitis attenuation, suggesting that miRNA-132 could be a promising anti-inflammatory approach in multiple sclerosis therapy (Hamza and Abdullah, 2013[31]). Shrestha et al. conducted miRNA expression profiling utilizing a microarray to identify miRNAs regulated by nicotine in PC12 cells (Shrestha et al., 2020[69]). They claimed that nicotine causes the expression of miRNA-132-5p to be induced (Shrestha et al., 2020[69]). The findings of this study demonstrated that activation of the nAChR promotes cell survival by increasing miRNA-132-5p, which in turn increases the anti-apoptotic protein Bcl-2 (Shrestha et al., 2020[69]). Similarly, another study showed that nicotine downregulates anti-inflammatory miRNAs in lung cells (Ebrahimpour et al., 2019[21]). Therefore, the relationship between nicotine as an activator of nAChRs, and increased expression of anti-inflammatory miRNAs is one of the possible molecular mechanisms to explain the anti-inflammatory effects of nicotine.

### miRNA-133

In some diseases, the disease causes abnormality in the expression of miRNAs independent of the patients' smoking status. MiRNA-133a-3p levels in lung cancer patients were independently linked with smoking, according to multivariate analysis (Ramírez-Salazar et al., 2021[60]). Significant correlations were found between this miRNA and many illnesses, such as lung cancer, inflammation, and pulmonary hypertension. The results emphasize how cigarette smoking impacts the reliable identification of circulating miRNAs as diagnostic biomarkers in lung cancer, and propose a smoking-dependent pathogenic function of miRNA-133a-3p in smokers (Ramírez-Salazar et al., 2021[60]).

### miRNA-140

This miRNA is known as nicotine-sensitive miRNA (Balaraman et al., 2012[4]; Solleti et al., 2017[71]). Huang and Li analyzed miRNA expression to investigate how much nicotine modifies miRNAs and tested the hypothesis that miRNAs could mitigate nicotine's impact on the regulation of gene expression. The findings showed that nicotine mediates the expression of miRNA-140, which targets the UTR region of dynamin-1 mRNA (Huang and Li, 2009[33]). They concluded that nicotine controls dynamin-1 expression via the miRNA-related pathway.

### miRNA-141

Studying the relationship between nicotine exposure and subsequent activation of nAChRs and changes in the expression of miRNAs provides future research directions in epigenetic studies related to nicotine exposure. In an experimental animal model, Faheem et al. looked at how nicotine affected the expression of miRNA-141 concerning metabolic diseases (Faheem et al., 2020[25]). Nicotine decreased glucose tolerance and significantly increased the expression of miRNA-141 in a dose-dependent manner. Nicotine produces a dose-response relationship between diabetes mellitus and insulin resistance, and miRNA-141 can be a promising biomarker for metabolic disorders (Faheem et al., 2020[25]).

### miRNA-155

Nicotine is associated with the progression of coronary atherosclerosis. Similarly, epigenetic alterations are widely recognized to be an essential factor contributing to atherosclerosis. Wang et al., by qRT-PCR analysis, showed a remarkable increase in miRNA-155 levels in extracellular vesicles of smoker patients, which was accompanied by worsening atherosclerosis (Wang et al., 2022[83]). Thus, miRNA-155-targeted therapy in nicotine related-atherosclerosis patients seems highly feasible, and the noninvasive blood test for circulating miRNA-155 would enable the correct detection of the disorders.

### miRNA-200

Epigenetic alterations such as changes in miRNAs expression profile play essential roles in the pathogenesis of diseases. Exposure to nicotine is associated with epigenetic modifications in numerous health conditions. In contrast to never smokers, Lei et al. showed that nicotine exposure decreases the expression of miRNA200c in colorectal cancer tissues (Lei et al., 2019[38]). Moreover, in a confirmatory *in vitro* study, nicotine suppressed miR‐200c expression in a dose‐ and time‐dependent manner in CRC cell lines.

### miRNA-218

Nicotine, an essential component of tobacco, is a significant risk factor for lung cancer, but the mechanism through which nicotine promotes lung cancer development remains unclear. Patients who smoked displayed reduced miRNA-218 expression. In lung cancer cells, nicotine reduced the expression of miRNA-218, according to Liu et al. (2019[43]). The miRNA-218- or nicotine-induced proliferative effects were rescued by the recovery of the expression level of CDK6, which indicated the role of the miRNA-218/CDK6 axis (Liu et al., 2019[43]).

### miRNA-221

Many studies were conducted to identify miRNAs specifically regulated explicitly by the nAChR agonist nicotine. After repeatedly administering nicotine to rats, Gomez et al. used a miRNA array test to assess miRNA expression in the prefrontal brain of the animals. The findings showed that after nicotine administration, miRNA-221 was significantly elevated (Gomez et al., 2016[26]). Nicotine-induced locomotor activity with alterations of phosphorylated extracellular signal-regulated kinase 1/2 activity. Understanding the molecular mechanism of nicotine-induced miRNA-221 expression may provide possibilities for targeted therapy with nAChR agonists.

## miRNA Clusters

### miRNA-199a/214

miRNA-dependent gene expression is designated as one of the mechanisms associated with smoking-induced disorders. Pittenger et al. focused on the effects of sex differences with nicotine intake and studied the miRNAs that were significantly and differentially altered by nicotine self-administration (Pittenger et al., 2018[54]). They found that the expression of miRNA cluster miRNA-199a and miRNA-214 upregulated in the female rats exposed to nicotine. The results indicated that miRNA-199/miRNA-214 upregulation via Sirtuin 1 may be associated with nicotine-induced effects in females and serve as a novel therapeutic target for sex-specific treatment approaches.

### miRNA-99a/192

Du et al. indicated that nicotine treatment induced tumor-associated properties by downregulation of miRNA-99b and miRNA-192 expression in lung cancer cell lines (Du et al., 2018[20]). Restoration of these miRNA's expression relieved the tumor-induced effects of nicotine. Further functional analysis revealed that miRNA-99b targets the downstream signaling molecules retinoblastoma one and fibroblast growth factor receptor three, respectively (Du et al., 2018[20]). This study provides new knowledge into the interpretation of underlying molecular mechanisms of nicotine-induced lung cancer.

## Anti-Inflammatory Activity of Nicotine Through miRNA Pathway

miRNAs regulate the expression of their targets to participate in multiple biological processes, such as inflammatory responses. miRNA-124 is an essential regulator of inflammatory gene expression (Xiao et al., 2016[89]; Yang et al., 2021[90]). Various studies showed changes in the expression level of miRNA-124 following nicotine treatment (Ulloa, 2013[81]). This miRNA, as one of the tumor suppressor miRNAs in most cancers, seems to have an essential relationship with the observed effects of nicotine (Sun et al., 2013[74]; Song et al., 2021[72]). The expression of miRNA-124 has decreased in some cancers (Roshani Asl et al., 2021[64]). This miRNA is important in managing inflammatory situations in addition to its function in malignancies (Qin et al., 2016[58]). Studies have been done on miRNA-124's significant function in the prevention of inflammatory disorders (Liang et al., 2020[40]; Han et al., 2021[32]; El Gazzar et al., 2022[23]). miRNA-124 mediates the function of cholinergic anti-inflammatory pathways (CAP) by inhibiting the production of pro-inflammatory cytokines (Sun et al., 2013[74]). This miRNA increased nearly four-fold in the presence of nicotine (Sun et al., 2013[74]). CAP is an intrinsic mechanism whereby the cholinergic signaling through the vagus nerve and the release of acetylcholine can lead to the inhibition of the release of inflammatory cytokines (Dhawan et al., 2012[17]). According to this inflammatory reflex concept, it is well-known that α7nAChR, which is expressed in immune cells such as macrophages, mediates CAP function (Tracey, 2007[79]; Dhawan et al., 2012[17]; Pinheiro et al., 2017[53]). Sun and colleagues confirmed that miRNA-124, by targeting STAT3, causes to suppression of inflammatory cytokines (Sun et al., 2013[74]). 

Similarly, Qin et al. studied the anti-inflammatory role of miRNA-124 and demonstrated that nicotine treatment enhanced miRNA-124 expression in an animal model of ulcerative colitis (Qin et al., 2017[57]). In this work, nicotine protects against ulcerative colitis by upregulating miRNA-124 and downregulating STAT3, suggesting that the miRNA-124/STAT3 axis may be a target for treating this condition (Qin et al., 2017[57]). In this regard, miRNA-124 may have potential therapeutic benefits in treating inflammatory diseases. Ulcerative colitis features a Th2-mediated response. Another functional study indicated that miRNA-124 protected against ulcerative colitis development with a Th1 polarization in a dextran sulfate sodium (DSS)-induced animal model of ulcerative colitis (Qin et al., 2020[59]). In their study, the results showed that the interleukin-6 receptor is a downstream target of miRNA-124 and could remarkably weaken the Th1 polarization induced by miRNA-124 (Qin et al., 2020[59]). Additionally, miRNA-132 has been demonstrated to have contributed to the regulation of inflammatory reactions, much to miRNA-124, which modulates the CAP (Priyadarshini et al., 2013[55]; Ulloa, 2013[81]). Liu et al. hypothesized that this miRNA might attenuate inflammation by enhancing the acetylcholine-mediated CAP (Liu et al., 2015[41]). They showed that miRNA-132 was upregulated after LPS supplementation in macrophages, and subsequently downregulated AChE protein via posttranscriptional regulation of the AChE enzyme. Reversely, the transfection of miRNA-132 mimic enhanced the ACh-mediated CAP by targeting AChE mRNA. Further studies reveal that miRNA-132 inhibits the nuclear translocation of NF-kB. They concluded that miRNA-132, by potentiating the CAP, exerts anti-inflammatory action in alveolar macrophages (Liu et al., 2015[41]).

## Early Life Nicotine Exposure and miRNA Expression

Maternal cigarette smoking is one of the well-known risk factors for disease in postnatal life, but the exact underlying mechanisms remain to be understood (Altıntaş et al., 2021[3]). Through the expression of miRNAs in offspring, numerous research examined the long-term consequences of maternal nicotine exposure on molecular and epigenetic pathways. For instance, maternal nicotine exposure induced higher miRNA-224 expression in offspring (Peixoto et al., 2021[51]). In a recent study to investigate the nicotine exposure-mediated effects, nicotine was administered to pregnant rats, and experiments were performed in offspring pups (Li et al., 2022[39]). The findings showed that maternal nicotine exposure significantly enhanced the expression of miRNA-181. These interesting findings provide a novel mechanism that aberrant alteration of the miRNA-181 plays a crucial role in perinatal nicotine exposure-mediated diseases in offspring (Li et al., 2022[39]). Additionally, research on the long-term effects of nicotine exposure has shown that smokers' spermatozoa have miRNA alterations, which may have an impact on how their future offspring develop (Bruin et al., 2010[6]; Marczylo et al., 2012[47]). Furthermore, several miRNAs (miRNA-16, miRNA-21, and miRNA-146a) are downregulated in the placenta of smoking mothers (Maccani et al., 2010[44]). Hence, maternal nicotine exposure during lactation induces obesity, thyroid, brown adipose tissue, and liver dysfunction in adult offspring. Both thyroid function and lipid metabolism are altered by the action of miRNAs (Maccani et al., 2010[44]). Several other studies emphasize the pivotal role of miRNAs in mediating the nicotine effects in offspring.

## Conclusions

miRNAs derived from endogenous transcripts are double-stranded RNA molecules at the length of about 22 nucleotides that do not encode proteins. These molecules are stable in circulating body fluids such as plasma or serum via the packaging into lipoprotein complexes such as exosomes and provide predictive information about the health status of the body and the possibility of developing diseases. A more significant number of miRNAs were altered in smokers' than in non-smokers' subjects. Rauthan et al. examined miRNA regulation of nAChR expression in a highly pertinent study (Rauthan et al., 2017[61]). They showed that the upregulation of nAChRs after exposure to nicotine is mediated by the miRNA-238-dependent pathway, and such upregulation is critical in the observed effects of nicotine (Rauthan et al., 2017[61]). Since the role of nicotinic receptors and their relationship with miRNAs in regulating the initiation and progression of diseases has remained unclear, it is hoped that by clarifying the role of these receptors and the signaling mechanisms associated with them, therapeutic strategies will be developed in the future. Smoking also extensively affects the expression of miRNAs in stem cells (SC). Previous studies indicated that cigarette smoking inhibits SC recruitment to tissues and delays the wound-healing process. Ng et al. studied the function of SC in the presence of nicotine and observed various changes in the function of these cells, including alteration in miRNA expression (Ng et al., 2013[50]). They concluded that miRNAs might play a pivotal role in the nicotine effects on SCs. Their research may provide plausible mechanical reasons for SC-related abnormalities in smoker participants as well as an explanation for why smoking slows down the healing processes connected with SC (Ng et al., 2013[50]). There will be a new development in the prevention and treatment of patients suffering from smoking-related diseases with the production of new drugs. Another study showed the effects of nicotine on the anti-tumor activity of immune cells. It showed that nicotine exhausted CD8^+^ T cells against tumor cells via increasing miRNA-629-5p to suppress interleukin-2 receptor subunit beta (Cheng et al., 2021[10]). Nicotine inhibits the tumor-suppressing ability of CD8^+^ T cells through the miRNA pathway. 

The cigarette smoke-induced alteration in miRNA expression in some tissues is close to those in adjacent tissues. For instance, the alterations in miRNA expression in the small airway, bronchial, and nasal tissues resembled each other (Sewer et al., 2020[66]). Some studies were performed to examine miRNAs expression profile. The analysis showed that some miRNAs change when exposed to cigarette smoke (e.g., miRNA-125b-5p, miRNA-132-3p, miRNA-99a-5p, and miRNA-146a-5p) (Sewer et al., 2020[66]). In addition to nicotine deregulation of miRNAs is caused by chronic exposure to other tobacco smoke-related N-Nitrosamines, such as 4-(methylnitrosamino)-1-(3-pyridyl)-1-butanone and N-Nitrosodiethylamine (Doukas et al., 2022[18]) (Figure 1B(Fig. 1)). These tobacco smoke components and nicotine could induce miRNA deregulation and are strongly linked to deregulated specific miRNAs, such as miRNA-21, miRNA-155, miRNA-34a, and miRNA-451a (Doukas et al., 2022[18]). The important cellular regulators nAChRs and miRNAs also control a wide range of genes involved in differentiation, proliferation or apoptosis, immune responses, and numerous other signaling pathways connected to these processes. It is hoped that by identifying the molecular mechanisms of the effects of nicotine in the causation and spread of diseases, new treatment methods can be established.

## Declaration

### Conflict of interest

The authors declare that there are no conflicts of interest.

### Statement of financial support

The grant of this study was provided by Stem Cell Research Center of Tabriz University of Medical Sciences, Tabriz, Iran (grant number: 71056) and conducted under ethical approval of Tabriz University of Medical Sciences Ethics Committee (ethical code: IR.TBZMED.VCR.REC.1401.066).

### Acknowledgment

The authors would like to thank the Stem Cell Research Center, Tabriz University of Medical Sciences for their support.

### Author contributions

K.H. and M.R.A. devised the main conceptual ideas. K.H. wrote the initial draft of the manuscript and prepared the table and figures. B.N., P.S., N.D., and M.R.A., reviewed the manuscript and edited it critically for important intellectual content. M.R.A. supervised the study. All of the authors have read and approved the final version submitted.

### Data availability

Data sharing not applicable to this article as no datasets were generated or analyzed during the current study.

## Figures and Tables

**Table 1:**
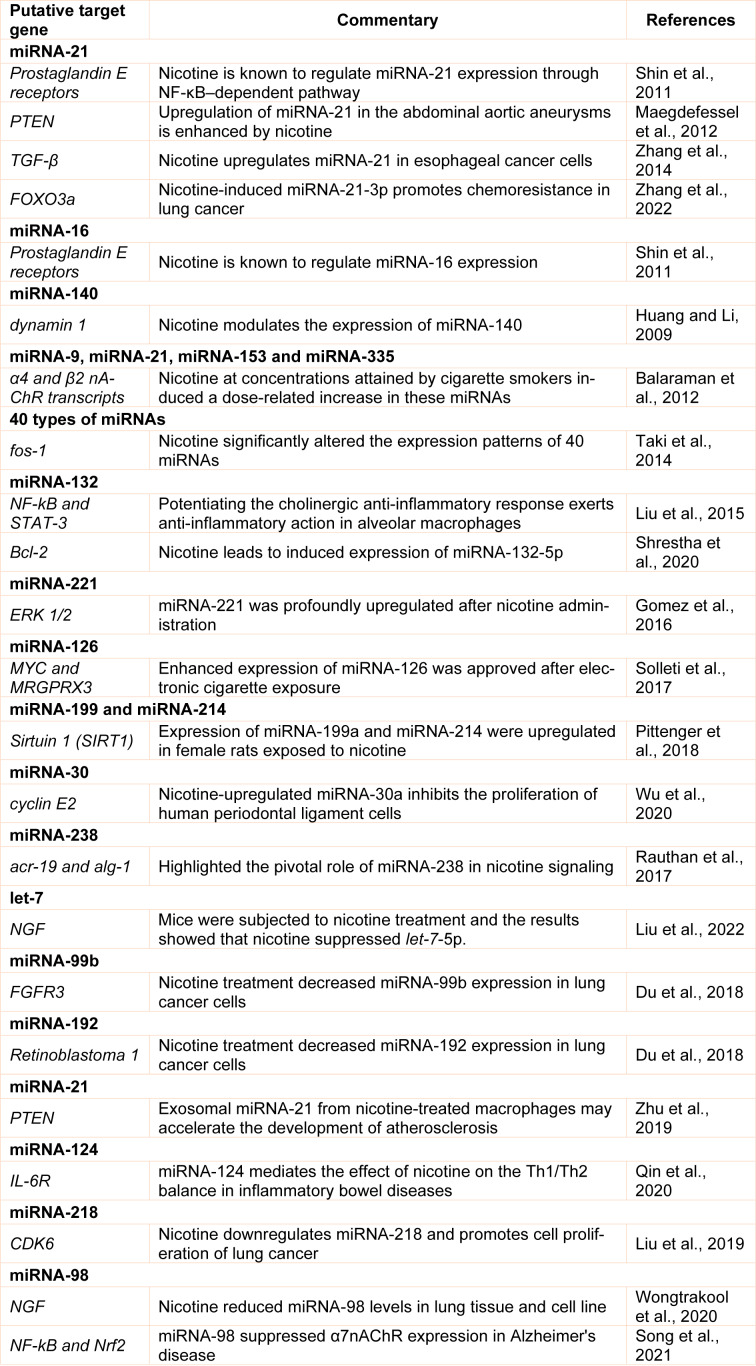
Summary of miRNAs modulated by nicotine and the activity of nicotinic receptors and subsequently change the expression of target genes
